# Connected Medical Technology and Cybersecurity Informed Consent: A New Paradigm

**DOI:** 10.2196/17612

**Published:** 2020-03-30

**Authors:** Jeffrey Tully, Andrea Coravos, Megan Doerr, Christian Dameff

**Affiliations:** 1 Department of Anesthesiology and Pain Medicine UC Davis Medical Center Sacramento, CA United States; 2 Elektra Labs Boston, MA United States; 3 Digital Medicine Society Boston, MA United States; 4 Harvard-MIT Center for Regulatory Science Boston, MA United States; 5 Policy Innovation Lab of Tomorrow Penn State University State College, PA United States; 6 Sage Bionetworks Seattle, WA United States; 7 Department of Emergency Medicine University of California San Diego La Jolla, CA United States; 8 Department of Computer Science and Engineering La Jolla, CA United States

**Keywords:** connected medical technology, cybersecurity, informed consent, privacy, patient autonomy, digital health, medical devices, ethics

## Abstract

**Background:**

Connected medical technology is increasingly prevalent and offers both a host of new therapeutic potentials and cybersecurity-related considerations. Current practice largely does not include discussions of cybersecurity issues when clinicians obtain informed consent.

**Objective:**

This paper aims to raise awareness about cybersecurity considerations for connected medical technology as they relate to informed consent discussions between patients and clinicians.

**Methods:**

Clinicians, health care cybersecurity researchers, and informed consent experts propose the concept of a cybersecurity informed consent for connected medical technology.

**Results:**

This viewpoint discusses concepts designed to facilitate further discussion on the need, development, and execution of cybersecurity informed consent.

**Conclusions:**

Cybersecurity informed consent may be a necessary component of informed consent practices, as connected medical technology proliferates in the health care environment.

The practice of medicine is built on the foundation of clinician-patient engagement, and consent is a key pillar supporting this essential relationship [1]. As medicine has shifted from a paternalistic, subordinating art to a collaborative effort of shared goal setting and decision making between parties, so too has the understanding of the ethics and acquisition of consent evolved.

It is widely acknowledged that consent must be *informed*. This charge demands that clinicians empower patients in shared decision making through culturally competent, plain language dialogue. In doing so, a patient’s informed consent becomes the embodiment of the principle of autonomy as well as a symbol of their investment as the most important stakeholder in the therapeutic alliance.

In the clinical realm, informed consent precedes care such as new treatment regimens or proposed surgical procedures. The discussion between healer and cared-for has come to possess a distinct anatomy. First, the patient is identified, which must involve not just a name and a record number, but the understanding of their personal story, beliefs, and objectives. Second the clinician discusses the nature of the intervention and presents a tailored list of benefits, risks, and alternatives to the stated plan. The persistent pace of progress in medicine ensures that these latter considerations are closely tied to new frontiers in clinical science.

Connected medical technology occupies one such frontier. From wearable activity trackers and mobile software apps to implantable medical devices and telemedicine platforms, digital tools are assuming an ever-growing role in health care, with an amplified potential depending on the degree these technologies connect to other devices, computers, or networks [2]. The acquisition of large amounts of increasingly granular data and the facilitation of longitudinal and remote clinical interactions are all enabled in part by the continuous connectivity of these devices.

Though the benefits of such connectivity in diagnosing, monitoring, and treating diseases are widely touted, connectivity may introduce additional risks to patients. As connected medical devices are built with the same or similar hardware and software used in mobile technologies and computers, flaws in code, components, or networks can lead to exploitation and disruption of these devices [3]. The analysis of and protection against such attacks constitute a central element of the practice of cybersecurity [4].

Researchers have demonstrated cybersecurity vulnerabilities in medical devices including automated internal cardioverter defibrillators, bedside infusion pumps, and implantable insulin delivery systems [5]. Such flaws, if abused, could lead to a number of consequences ranging from exposure of personal and private health information to the malfunction of devices resulting in physical harm. Though there are not yet any reports of patients directly affected by the exploitation of a medical device’s cybersecurity vulnerability, the potential for such events has led to concerted efforts from manufacturers, regulators, and security professionals to advocate for and improve medical cybersecurity practices.

Clinicians are expected to acquire knowledge on various medications, procedures, and therapies to understand and articulate the risks, benefits, and alternatives of such interventions during the informed consent process. As an increasing number of interventions rely solely or in part on connected technologies, the same attendant framework should exist for these tools. We propose that the unique characteristics of connected technologies warrant development of a “cybersecurity-informed consent” to address the cybersecurity implications of planned interventions. Several challenges exist in creating a model for such a consent.

First, the epidemiology of health care cybersecurity is an emerging science. The benefits of connected medical devices have been quickly embraced; however, cybersecurity itself has been a blind spot for many practicing connected medicine. This disinclination may arise from a lack of knowledge or understanding of cybersecurity vulnerabilities, or may relate to the lack of identified real-world incidents of cybersecurity vulnerabilities interfering with clinical care. However, the US Food and Drug Administration, in its authority as primary regulator of medical devices, has issued multiple safety communications and recalls consistent with the maxim that “absence of evidence is not evidence of absence” [6-8].

A second challenge is the risk-benefit ratio of addressing cybersecurity vulnerabilities. One of the most basic remedies for cybersecurity vulnerabilities is the practice of “patching”—updating a device’s software with new and improved code to address flaws. This is usually a relatively straightforward exercise, occurring almost continuously in many enterprises that use commercial operating systems. However, patching has added complexity with medical devices, because a small but nonnegligible risk exists when software updates intended to address cybersecurity dangers lead to unintended corruption of the device’s normal functioning. Furthermore, while the risk of exploitation of a vulnerability may be unknown, the failure rate of a patch may be well-documented [9]. This risk-to-reward imbalance may result in some clinicians advising patients to forego patching of vulnerabilities entirely.

Third, in contrast to much of medicine, the cybersecurity risk of connected medical interventions may not be fixed—or even consistent—across the life span of a device or app used in an intervention. The same properties in software that allow for updates in functionality and features ensure that the potential for new vulnerabilities exist alongside the promise of additional benefits. A clinician accustomed to largely stable probabilities of, for example, infection with transfusion of blood products may confront a situation with a connected device where current cybersecurity risk is undefined and future danger remains amorphous.

Given the increasing ubiquity of networked functionality in digital devices ranging from the smallest wearables to the largest surgical robots, the simple option of using nonconnected technologies may not be a choice for clinicians or patients concerned about cybersecurity risks. Those looking to benefit from the most sophisticated pacemakers or insulin pumps may face the realization that alternatives to these devices might not exist.

The traditional model of clinician-facilitated informed consent relies on the previously discussed core knowledge that many clinicians currently lack. There are increasing efforts to raise awareness of the potential consequences of cybersecurity vulnerabilities on patient safety; however, there is still no widespread curricula for allied health professional students or concerted continuing medical education for practicing providers. Without at least a basic, conceptual understanding of both the risks and remedies, it is both unfair and impractical to expect the clinician to impart the same information for patient consideration.

Yet patients surely deserve the chance to consider cybersecurity elements within the context of their treatment plans or procedures, and cybersecurity-informed consent creates the environment for the patient to be an active participant in this process. The challenge then becomes incorporating cybersecurity-informed consent into a complex workflow process that already demands significant time and effort, which is largely uncompensated. Several potential models may warrant further evaluation.

The highest yield method for cyber-informed consent adoption may be educational interventions for the providers whom are most likely to interface with connected medical technologies, including electrophysiologists, endocrinologists, and informaticians, in the form of digital modules, tool kits, or simulations.

An argument may be made that, as cybersecurity concepts are not immediately germane to the practice of medicine, there exists no explicit demand for clinician involvement in the consent process. This framework implies that the obligation to cyber-consent belongs to other stakeholders such as the manufacturers of the technology. In such a system, a patient might interface with a particular vendor to be educated about the cybersecurity implications of a device in a similar way that proceduralists rely on device representatives to provide technical guidance during the implantation process.

Informed consent methodology in clinical research has recently evolved in a number of interesting ways that may serve as a good model for cyber-informed consent.

The opportunity to obtain consent remotely through online portals, along with the ability to present a wide variety of information customized to individual capabilities and cultures, has increased inclusivity and interactivity. It has also generated the idea of “independently navigable” consents that patients complete autonomously without requiring direct interaction with the research team [10,11].

It is clear that more research is needed to determine which cybersecurity-informed consent approach best addresses the unique hurdles of digital medicine. Having a clear, concise informed consent process can be challenging in light of clinician inexperience, as well as uncertainty regarding the true level of risk posed to patients by connected technologies (and the potential for these risks to fluctuate over time). As connected medical interventions offer personalization of care and expanded accessibility, so too will an effective cyber-informed consent empower clinicians and patients in goal-affirming health care decision making.
